# Hot Deformation Characteristics and Dynamic Recrystallization Mechanisms of a Semi-Solid Forged AZ91D Magnesium Alloy

**DOI:** 10.3390/ma17163939

**Published:** 2024-08-08

**Authors:** Zehua Yan, Guozheng Zhang, Sheng Yang, Wei Zhang, Huiyan Ning, Bo Xu

**Affiliations:** 1 Rongcheng College, Harbin University of Science and Technology, Weihai 264300, China; yzh@hrbust.edu.cn (Z.Y.); zz411583141@163.com (G.Z.); hanjiyangsheng@126.com (S.Y.); zhangwei8171@hrbust.edu.cn (W.Z.); 2 School of Mechanical and Electrical Engineering, Heilongjiang Institute of Technology, Harbin 150050, China; m13679328401@163.com

**Keywords:** semi-solid forged AZ91D magnesium alloy, hot compression, constitutive equation, microstructure evolution, dynamic recrystallization

## Abstract

Magnesium alloys show great promise in high-speed transport, aerospace, and military technology; however, their widespread adoption encounters challenges attributed to limitations such as poor plasticity and strength. This study examines the high-temperature deformation of semi-solid forged AZ91D magnesium alloy through a combination of experiments and simulations, with a focus on comprehending the influence of deformation conditions on dynamic recrystallization (DRX). The findings disclose that conspicuous signs of DRX manifest in the yield stress curve as strain increases. Additionally, decreasing the strain rate and temperature correlates with a reduction in both yield stress and peak strain, and the activation energy is 156.814 kJ/mol, while the critical strain and peak strain remain relatively consistent ($\varepsilon_{c}=0.66208\varepsilon_{p}$). Microstructural changes during high-temperature deformation and the onset of DRX are thoroughly examined through experimental methods. Moreover, a critical strain model for DRX and a predictive model for the volume fraction of DRX were formulated. These equations and models, validated through a combination of experiments and simulations, serve as invaluable tools for predicting the mechanical behavior and microstructural evolution, which also establishes a foundation for accurately predicting the deformation behavior of this alloy. By analyzing the hot deformation characteristics and dynamic compression mechanism of the newly developed semi-solid forging AZ91D magnesium alloy, a numerical simulation model can be effectively established. This model objectively reflects the changes and distributions of stress, strain, and rheological velocity, providing a scientific basis for selecting subsequent plastic deformation process parameters and designing mold structures.

## 1. Introduction

Magnesium and its alloys are widely utilized in industries such as automotive, aircraft, electronics, energy, and biomedical sectors due to their impressive attributes. These include excellent damping properties, specific stiffness, specific strength, specific rigidity, and ease of processing [1,2,3,4]. However, challenges arise from the hexagonal close-packed structure, limited room-temperature plasticity, and constraints in processing techniques, corrosion resistance, and cost [5,6,7]. Thermal processing above the recrystallization temperature is required in magnesium alloy production, influenced by factors like temperature, strain rate, and deformation, leading to a complex recrystallization process. Scholars focus on the hot deformation behavior and constitutive equations, like the Johnson–Cook model [8], Khan–Huang–Liang model [9], Fields–Backofen model [10], Voce–Kocks model [11], and Arrhenius model [12]. The hyperbolic sinusoidal form of the Arrhenius equation, widely used in materials research, is introduced by Sellars and Tagert [12]. Duan’s study investigates the flow behavior and microstructure of AZ80A magnesium alloy, exploring the influence of deformation temperature and strain rate on dynamic recrystallization (DRX) structure evolution and strain rate sensitivity index [13]. Luan conducts isothermal compression tests on AZ31 magnesium alloy to elucidate its deformation characteristics, indicating dynamic flow softening with accurate constitutive model predictions matching observed values [14]. Chen analyzes the microstructure and mechanical properties of Mg-Y-Zn alloy, developing processing charts for optimizing hot working and structural control, with predicted outcomes closely matching actual results [15]. Additionally, DRX is a vital mechanism for grain refinement and softening during deformation, such as forging, extrusion, stamping, and rolling. Research on magnesium and its alloys has identified various DRX mechanisms, including low-temperature dynamic recrystallization (LTDRX), discontinuous dynamic recrystallization (DDRX), continuous dynamic recrystallization (CDRX), twinned dynamic recrystallization (TDRX), and rotational DRX (RRX). Galiyev’s study revealed LTDRX in Mg-5.8Zn-0.65Zr alloys, showing a significant grain size reduction and increased hardness at 423 K and a strain rate of 2.8 × 10^−3^ s^−1^ [16]. Tan explored CDRX nucleation mechanisms in Mg-9Al-Zn alloy after high-temperature extrusion and torsion experiments [17]. DDRX research highlighted distinct nucleation and grain growth stages [18]. The research indicates that deformation temperature and strain rate largely determine the sufficiency of dynamic recrystallization in magnesium alloys. By selecting appropriate deformation temperatures and strain rates, different types of DRX can be induced, which eliminates stress concentration caused by work hardening and reduces the force required during the processing of magnesium alloys. However, investigations into DRX in magnesium alloys are limited, warranting further exploration. Establishing a correlation between the material’s constitutive model and DRX mechanisms is crucial for understanding deformation behavior.

It is well known that proper setting of process parameters such as extrusion, rolling, and forging can induce DRX and grain refinement in magnesium alloys, thus enhancing their microstructure and mechanical properties [19]. However, these conventional plastic deformation methods have several shortcomings. For instance, rolling alloy plates tend to form a strong basal plane texture, which hinders the secondary processing of deformed magnesium alloys and limits their applications [20]. The extrusion process tends to form a ring-like basal plane texture, leading to significant anisotropy in the alloy [21]. Additionally, the internal DRX is often incomplete, resulting in coarse uncrystallized regions and an uneven grain size distribution. This affects the material’s overall performance and results in high waste and a low finished product rate. Although it has been reported that conventional extrusion processes can refine the grain, the extent of grain refinement is generally limited. The development of severe plastic deformation (SPD) technology can further refine the grain size of the material, producing ultra-fine-grained materials [22]. However, SPD is highly complex, involving more processing stages and steps. Moreover, SPD technology imposes strict size limitations on the processed materials, restricting its application to smaller workpieces, primarily in laboratory settings, thus preventing large-scale industrial applications. To address the shortcomings of conventional machining processes and SPD technology, a new process combining pre-deformation with extrusion has been proposed. Jiang et al. [23] studied the Mg-1.58Zn-0.52Gd (wt.%) alloy by applying hot pre-forging at 350 °C and 400 °C followed by slow extrusion. They found that the extruded alloys without pre-forging exhibited a bimodal microstructure consisting of fine DRXed grains and coarse unrecrystallized grains with a strong basal texture. After pre-forging, the extruded alloys exhibited a finer, fully DRXed microstructure and a weaker extruded texture. Compared to the extruded non-wrought alloys, the pre-wrought alloys exhibited an excellent combination of properties in terms of strength, ductility, and yield asymmetry. This predeformation process generates a large number of twins, subgrains, and other defect structures in magnesium alloy ingots. These defects provide additional nucleation sites for DRX during subsequent extrusion, promoting recrystallization nucleation and grain refinement, thereby improving the alloy’s strength and plasticity.

Pre-deformation combined with subsequent plastic deformation is simpler than the intense plastic deformation method and can produce finer grains than conventional extrusion, improving the alloy’s tensile asymmetry [24]. However, after pre-forging, the ingot must be reheated. During reheating, the ingot’s internal twins and other defects can diminish, leading to grain growth and reducing the effectiveness of the pre-deformation. To maximize the benefits of pre-deformation, it is necessary to explore the metal forming processes following pre-deformation, particularly semi-solid die forging. This process can eliminate the need for a second preheating between pre-forging and extrusion, and it can significantly enhance the molding capability of the semi-solid billet. Considering the high concentration of the second phase within the alloy and its complex structure [25], it is imperative to investigate the thermal deformation and DRX behavior of semi-solid forged AZ91D magnesium alloy to construct a comprehensive material model, encompassing dynamic recovery and a recrystallization rate model tailored to DRX conditions. By analyzing the hot deformation characteristics and dynamic compression mechanism of the newly developed semi-solid forging AZ91D magnesium alloy, a numerical simulation model can be effectively established. This model objectively reflects the changes and distributions of stress, strain, and rheological velocity, providing a scientific basis for selecting subsequent plastic deformation process parameters and designing mold structures. Therefore, it is paramount to research and develop novel magnesium alloy forming methods, investigate the organizational evolution of fabricated parts, establish an intrinsic model, and lay a solid foundation for subsequent simulation research, thereby advancing the application of magnesium alloys across diverse fields.

## 2. Experimental Materials and Methods

### 2.1. Experimental Material

**Figure 1:**
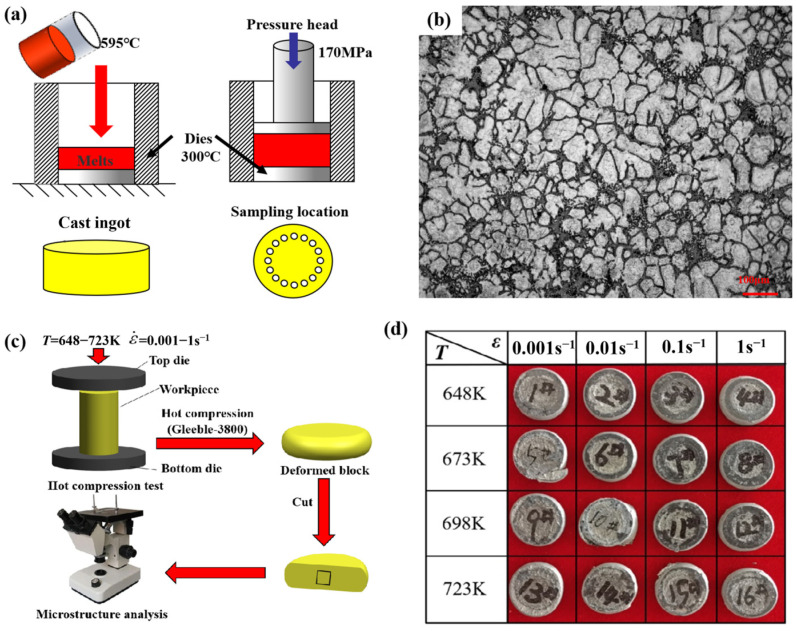
Schematic diagram of casting AZ91D magnesium alloy (**a**) and its metallography (**b**); schematic diagram of compressed alloys (**c**) and its macro-structure (**d**).

The AZ91D magnesium alloy, detailed in Table 1, was produced through semi-solid forging. The melting equipment used for metal casting in this experiment is a custom-built well-type resistance melting furnace (branch torch furance, Luoyang, China). The heating power is 15 kW, the rated temperature is 1000 °C, and the heating rate is 20 °C/min. Additionally, the furnace cover has an open mouth, facilitating easy transport and casting of molten metal. Furthermore, the furnace cover is equipped with a gas inlet to maintain a protective gas atmosphere throughout the melting process. Specifically, the furnace is filled with a mixture of CO_2_ and SF_6_ gas in a volume fraction ratio of 99:1. During the melting process, a graphite crucible with a 1 kg capacity is used for transfer metal melt. The casting mold, made of H13 steel with a preheating temperature of 300 °C, is coated with water-based graphite as a release agent and has a diameter of 60 mm with a thickness of 30 mm. The melting temperature is set to 750 °C. After the alloy in the crucible is fully melted, the temperature is maintained for 15 min before cooling. As illustrated in Figure 1a, the melt is allowed to cool to 595 °C before being quickly poured. After pouring, the melt is immediately forged under 170 MPa pressure for 3 min. Finally, after the casting is cooled, it is removed and a sample with a diameter of 8 mm and a height of 12 mm is cut from the mid-height of the casting for compression testing. Using a Gleeble-3800 thermal simulation tester (Poestenkill, NY, USA), the hot compression experiments were conducted with the specimen size of Φ6 mm × 9 mm at temperatures of 648 K, 673 K, 698 K, and 723 K, with compression rates of 0.001 s^−1^, 0.01 s^−1^, 0.1 s^−1^, and 1s^−1^. Post-compression, specimens (Figure 1c,d) were sectioned along their central axis, meticulously polished, and treated with a 4% nitric acid solution for corrosion. Transmission electron microscopes (TEM, JEOL JEM-2100F, Tokyo, Japan) were also used to characterize the microstructures in this work.

### 2.2. Finite Element Models

As AZ91D magnesium alloy was not available in the Deform-3D material library, the derived equations were added to create the AZ91D material profile. Physical parameters from data sources [12], detailed in Table 2 and measured in SI units, were integrated. For the finite element model in hot compression, the workpiece was considered plastic, and the upper and lower indenters were treated as rigid. Meshing was applied only to the workpiece with a grid resolution of 30,000 elements. To simulate isothermal compression, the workpiece, indenter, and surroundings were adjusted to match experimental conditions.

## 3. Results and Discussion

### 3.1. Stress–Strain Curve

**Figure 2:**
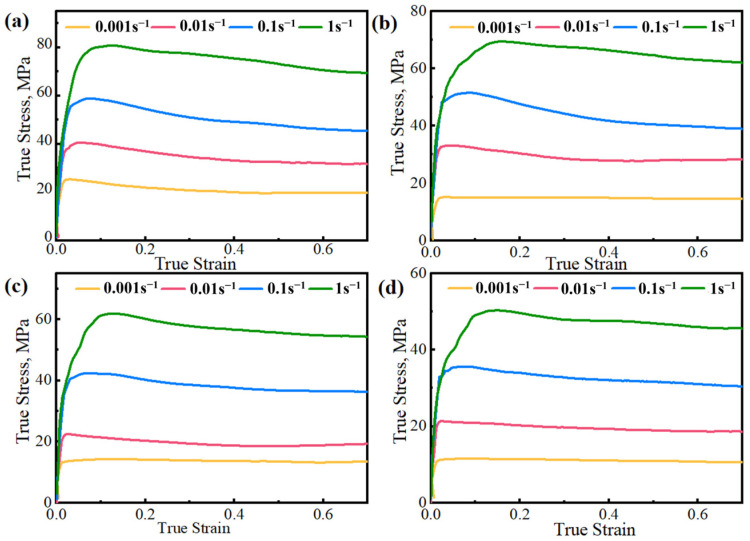
Stress–strain curves of AZ91D magnesium alloy: (**a**) 648 K; (**b**) 673 K; (**c**) 698 K; (**d**) 723 K.

Figure 2 displays the stress–strain curve of AZ91D magnesium alloy, exhibiting a characteristic DRX curve. The curve indicates an increase in flow stress with higher strain rates and lower deformation temperatures. As deformation continues, the flow stress rises significantly, reaching a peak value. Subsequently, it decreases beyond the peak, stabilizing at higher temperatures or lower strain rates. This highlights the heightened sensitivity of AZ91D magnesium alloy’s flow stress to variations in strain rate and deformation temperature during hot compression.

### 3.2. Constitutive Equation

Throughout the deformation, where the interplay between work hardening and softening occurs, the Garofalo creep equation emerges as a precise descriptor for the relationship between flow stress (*σ*), strain rate ($\dot{\varepsilon }$), and temperature (*T*) [26,27,28]:

(1) $$\left\{\begin{array}{l} \dot{\varepsilon }=A_{1}\sigma^{n_{1}}\exp\left(-\frac{Q}{RT}\right)\;\mathrm{all}\;\alpha \sigma \;\\ \dot{\varepsilon }=A_{2}\exp\left(\beta \sigma \right)\;\alpha \sigma <0.8\\ \dot{\varepsilon }=A{\left[\sinh\left(\alpha \sigma \right)\right]}^{n}\;\alpha \sigma >1.2 \end{array}\right.$$

where *A*_1_, *A*_2_, *A*, *α*, *β*, *n*, *n*_1_ are the material parameters; *α* for the stress level parameter, *α* = *β*/*n*_1_; *A* is the structure factor; *n* is the stress index; *Q* is the activation energy; *R* is the standard molar gas constant (8.314 J/mol/K). 

The logarithmic treatment of Equation (1) yields:

(2) $$\left\{\begin{array}{l} \ln\dot{\varepsilon }=\ln A_{1}+n_{1}\ln\sigma -\frac{Q}{RT}\\ \ln\dot{\varepsilon }=\ln A_{2}+\beta \sigma -\frac{Q}{RT}\\ \ln\dot{\varepsilon }=\ln A+n\sinh(\alpha \sigma )-\frac{Q}{RT} \end{array}\right.$$

**Figure 3:**
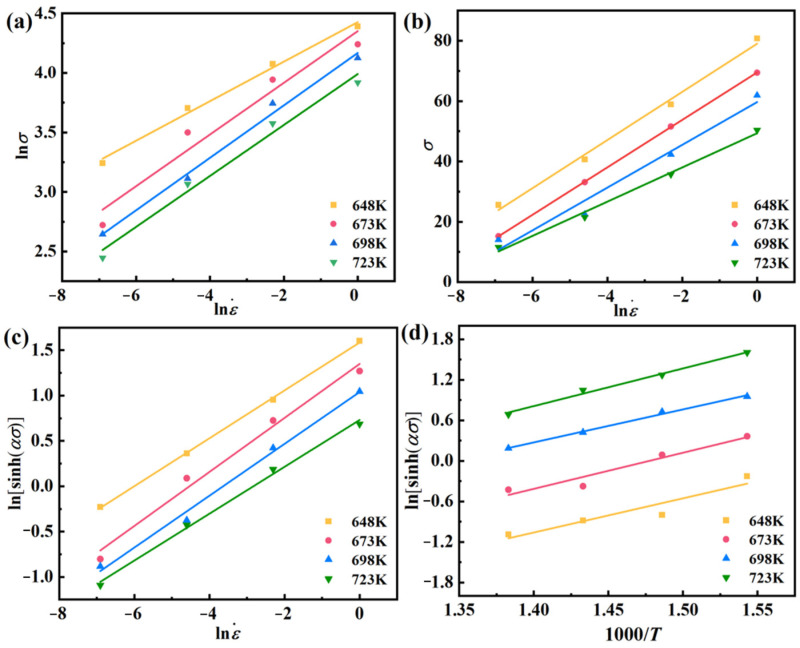
Linear fit between (**a**) ln*σ* − ln$\dot{\varepsilon }$, (**b**) *σ* − ln$\dot{\varepsilon }$, (**c**) ln$\dot{\varepsilon }$ − ln[sinh(*ασ*)], (**d**) ln[sinh(*ασ*)] − 1000/*T*.

Per McQueen’s findings [29], the peak stress (*σ*_p_) is predominantly chosen for establishing their constitutive equations. As depicted in Figure 3, there is a distinct linear correlation between ln$\dot{\varepsilon }$ and ln*σ*, ln$\dot{\varepsilon }$ and *σ*, ln$\dot{\varepsilon }$ − ln[sinh(*ασ*)], ln[sinh(*ασ*)], and 1000/*T*, so one can obtain Equation (3) from Equation (2):

(3) $$\left\{\begin{array}{l} n_{1}={\left.\frac{\partial \left(\ln\dot{\varepsilon }\right)}{\partial \left(\ln\sigma \right)}\right|}_{T}\\ \beta ={\left.\frac{\partial \left(\ln\dot{\varepsilon }\right)}{\partial \sigma }\right|}_{T}\\ n={\left.\frac{\partial \left(\ln\dot{\varepsilon }\right)}{\partial \left(\ln\left[\sinh\left(\alpha \sigma \right)\right]\right)}\right|}_{T}\\ Q={\left.Rn\frac{\partial \left(\ln\left[\sinh\left(\alpha \sigma \right)\right]\right)}{\partial \left(1/T\right)}\right|}_{\dot{\varepsilon }} \end{array}\right.$$

Combining Figure 3 and Equation (3), one can obtain *n*_1_ is 4.89446, *β* is 0.13982, *α* (=*β/n*_1_) is 0.02857 MPa^−1^, *n* is 3.61637, ∂ln[sinh(*ασ*)]/∂(1/*T*) is 5.21557, so *Q* is 156.814 KJ/mol. And the Arrhenius constitutive expression can be derived:

(4) $$\dot{\varepsilon }=1.33208\times 10^{10}{\left[\sinh\left(0.02857\sigma \right)\right]}^{3.61637}\exp\left(156814/RT\right)$$

Z-parameter is a convenient way of describing the effect of strain temperature and strain rate during the forming process [12,30,31,32].

(5) $$Z=\dot{\varepsilon }\exp\left(\frac{Q}{RT}\right)$$

Bringing Equation (5) into Equation (1) and taking its logarithms, one can obtain Equation (6):

(6) $$\ln Z=\ln A+n\ln\left[\sinh\left(\alpha \sigma \right)\right]$$

**Figure 4:**
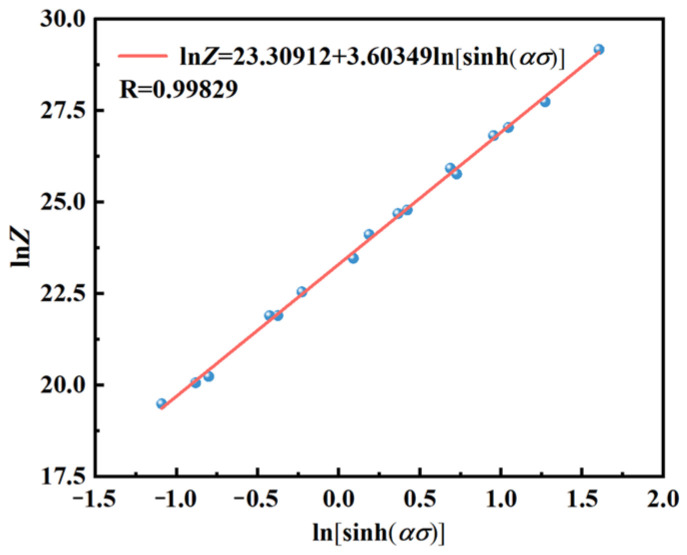
ln*Z* − ln[sinh(*ασ*)] linear fitting plot.

Figure 4 demonstrates a strong linear relationship between ln*Z* and ln[sinh(*ασ*)], the *n*-value (3.60349) differing by only 0.01288 from the computed (3.61637); this minimal discrepancy corresponds to an error rate of no more than 0.36%, affirming the precision and reliability of using the *Z*-parameter as a reflection of its σ.

According to Equation (6), the σ can be expressed as Equation (7):

(7) $$\sigma =\ln\left\{{\left(\frac{Z}{A}\right)}^{1/n}+{\left[{\left(\frac{Z}{A}\right)}^{2/n}+1\right]}^{1/2}\right\}/\alpha$$

Bringing the data into Equation (8) yields the improved Arrhenius equation:

(8) $$\sigma =\frac{1}{0.02857}\ln\left\{{\left(\frac{Z}{1.33208\times 10^{10}}\right)}^{\frac{1}{3.61637}}+{\left[{\left(\frac{Z}{1.33208\times 10^{10}}\right)}^{\frac{2}{3.61637}}+1\right]}^{\frac{1}{2}}\right\}$$

Which:

(9) $$Z=\dot{\varepsilon }\exp\left(156814/RT\right)$$

### 3.3. Critical Condition Model for DRX

DRX involves the formation and growth of recrystallized grains in metallic materials, transpiring under favorable deformation conditions—comprising temperature and strain rate. Hence, meticulous control over these parameters proves crucial in attaining the targeted grain size and its associated properties. Therefore, it is necessary to understand the critical condition model for DRX. 

#### 3.3.1. Critical Condition Model for DRX

Per the Sellars model, the critical condition model for DRX integrates both peak and critical strain models [33,34]. Model expression for peak strain in the Sellars model:

(10) $$\varepsilon_{p}=ad_{0}^{n}Z^{m}$$

(11) $$Z=\dot{\varepsilon }\exp\left(Q/RT\right)$$

where *ε_p_* is the peak strain, *d*_0_ is the initial grain size. Taking Equation (11) into Equation (10), the peak strain model can also be expressed as:

(12) $$\varepsilon_{p}=a_{1}d_{0}^{n_{1}}{\dot{\varepsilon }}^{m_{1}}\exp\left(Q_{1}/RT\right)+C_{1}$$

where *Q*_1_ is the deformation activation energy; *a*_1_, *n*_1_, *m*_1_, *C*_1_ is a material-related parameter.

Since the internal grain sizes of AZ91D magnesium alloy are 57.8 µm, so the effect of *d*_0_ can be neglected. And Equation (12) can be expressed as Equation (13):

(13) $$\left\{\begin{array}{l} \varepsilon_{p}=a_{1}{\dot{\varepsilon }}^{m}\exp\left(Q_{1}/RT\right)\\ \ln\varepsilon_{p}=\ln a_{1}+m_{1}\ln\dot{\varepsilon }+Q_{1}/RT \end{array}\right.$$

**Figure 5:**
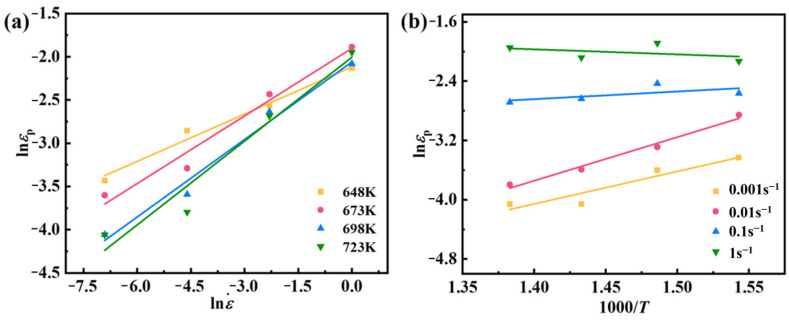
Linear fit between peak stress and deformation parameters: (**a**) ln*ε_p_* − ln$\dot{\varepsilon }$, (**b**) ln*ε_p_* − 1000/*T*.

From Equation (13), Figure 5a,b, Equation (14) can be obtained:

(14) $$\left\{\begin{array}{l} Q_{1}=R\frac{\partial \ln\varepsilon_{p}}{\partial \left(1000/T\right)}\\ m_{1}=\frac{\partial \ln\varepsilon_{p}}{\partial \ln\dot{\varepsilon }} \end{array}\right.$$

From Figure 5, one can get *m*_1_ = 0.2662, *Q*_1_ = 20,514.87814 J/mol, and *a*_1_ = 0.004569, so the peak strain model of AZ91D magnesium alloy can be obtained:

(15) $$\varepsilon_{p}=0.004569{\dot{\varepsilon }}^{0.2662}\exp\left(20514.87814/RT\right)$$

#### 3.3.2. Critical Strain Model

Jonas’ findings propose that the onset of DRX modifies the internal thermodynamic system of the alloy [35]. In the results, an inflection point appears in the *θ*–*σ* curve, indicating the critical conditions for DRX initiation. The expression for the work-hardening rate is outlined below:

(16) $$\theta =\frac{d\sigma }{d\varepsilon }=\frac{\Delta \sigma }{\Delta \varepsilon }$$

**Figure 6:**
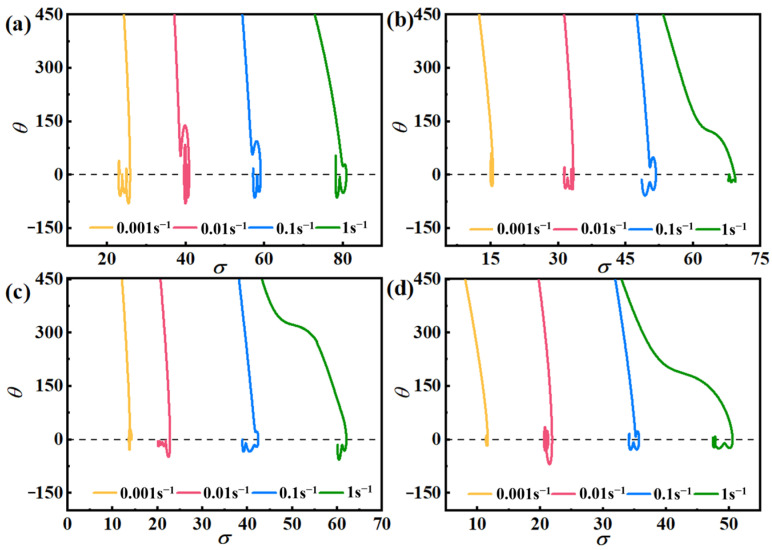
*θ–σ* curves of AZ91D Mg alloy at different temperatures: (**a**) 648 K, (**b**) 673 K, (**c**) 698 K, (**d**) 723 K.

Figure 6 depicts a notable decrease in *θ* as *σ* values rise, marked by a distinct inflection point in the curve, indicating the initiation of the DRX process. Concurrently, the corresponding σ-value represents the critical condition for DRX. 

Jonas and Najafizadeh confirmed that the vicinity of the inflection point of the *θ–σ* curve can be characterized by a third-order polynomial [35]:

(17) $$\theta =A_{1}\sigma^{3}+A_{2}\sigma^{2}+A_{3}\sigma^{1}+B$$

where *A*_1_, *A*_2_, *A*_3_, *B* are fitting constants. So:

(18) $$\sigma_{c}=-\frac{A_{2}}{3A_{1}}$$

**Figure 7:**
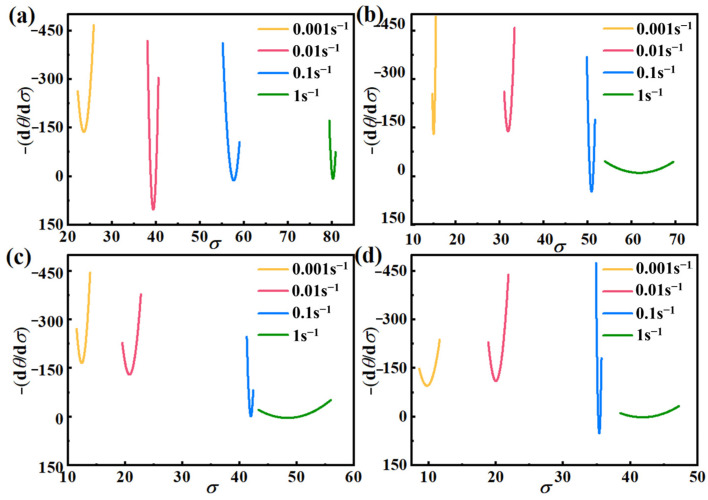
−(d*θ*/d*σ*) − *σ* curves of AZ91D magnesium alloy at different temperatures: (**a**) 648 K, (**b**) 673 K, (**c**) 698 K, (**d**) 723 K.

From Equation (18), one can see that −(∂*θ*/∂*σ*) has a quadratic relationship, and combined with Figure 6, the −(d*θ*/d*σ*) − *σ* curve is made, which is shown in Figure 7.

**Figure 8:**
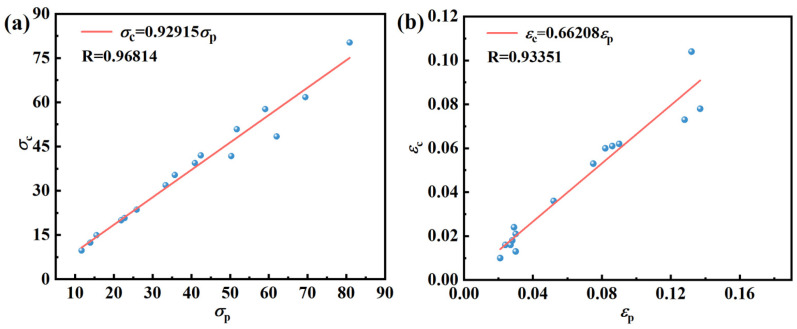
Linear fit between critical stress and strain and peak stress and strain (**a**) *σ*_c_ − *σ*_p_, (**b**) *ε*_c_ − *ε*_p_.

According to Figure 7, the *σ*_c_, *σ*_p_, *ε*_c_, and *ε*_p_ for different deformation conditions can be obtained, and the *σ*_c_ − *σ*_p_ linear fit and *ε*_c_ − *ε*_p_ linear fit are plotted in Figure 8. It is evident that the two fits align perfectly, aligning with the Sellars model’s principle that the ratio of *ε*_c_ to *ε*_p_ should fall within the range of 0.6 to 0.95. In this study, the relationship equation between *σ*_c_ and *σ*_p_, *ε*_c_ and *ε*_p_ can be expressed as Equation (19).

(19) $$\left\{\begin{array}{l} \sigma_{c}=0.92915\sigma_{p}\\ \varepsilon_{c}=0.66208\varepsilon_{p} \end{array}\right.$$

#### 3.3.3. DRX Kinetic Model

Currently, the widely employed DRX kinetic model, based on the Avrami equation, remains a prominent choice. In numerous hot simulation experiments, time is introduced as a variable to formulate the following DRX kinetic model [36]:

(20) $$X_{drx}=1-\exp\left(-xt^{y}\right)$$

Yada modified DRX’s kinetic model by replacing time with strain [37].

(21) $$X_{drx}=1-\exp\left[-x\left(f{\left(\varepsilon \right)}^{z}\right)\right]$$

where *X_drx_* is the percentage of DRX, *x* and *z* are material-related parameters, and *f*(*ε*) is a strain-related quantity. Furthermore, the DRX kinetics model can be expressed as:

(22) $$X_{drx}=1-\exp\left[-\beta_{d}{\left(\frac{\varepsilon -a_{10}\varepsilon_{p}}{\varepsilon_{0.5}}\right)}^{k_{d}}\right]$$

(23) $$\varepsilon_{0.5}=a_{5}d_{0}^{h_{5}}\varepsilon^{n_{5}}{\dot{\varepsilon }}^{m_{5}}\exp\left(Q_{5}/RT\right)+c_{5}$$

where X*_drx_* is the volume fraction of DRX, *Q*_5_ is the thermodynamic activation energy of DRX, $\dot{\varepsilon }$ is the strain rate, *ε*_0.5_ is the strain corresponding to 50% of DRX, and *β*_d_, *a*_10_, *k*_d_, *h*_5_, *m*_5_, and *c*_5_ are material correlation coefficients.

Taking logarithms of Equation (22) and writing *a*_10_*ε*_p_ as *ε*_c_, one can obtain Equation (24):

(24) $$\ln\left[-\ln\left(1-X_{drx}\right)\right]=\ln\beta_{d}+k_{d}\ln\frac{\varepsilon -\varepsilon_{c}}{\varepsilon_{0.5}}$$

**Figure 9:**
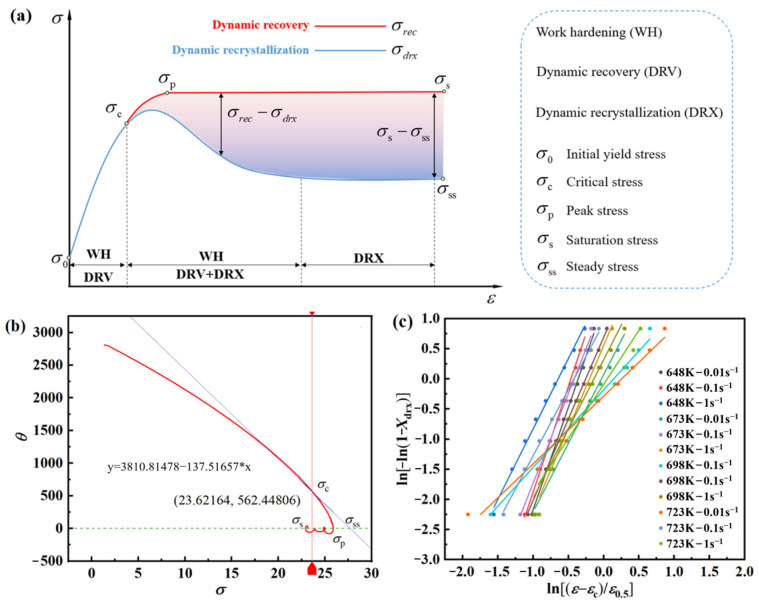
Characteristic curve: (**a**) analysis diagram (**b**) real stress–strain curve solved stress curve with 648 K/0.001 s^−1^; (**c**) ln[−ln(1 − *X_drx_*)] − ln[(*ε* − *ε*_c_)/*ε*_0.5_] linear fitting plots.

Figure 9a illustrates that DRX occurs after the material reaches σ_p_, and the curve descends to the steady-state stress σ_ss_, indicating material softening. Here, the degree of stress reduction from σ_p_ is denoted as σ_p_ − σ_exp_, and the maximum achievable softening of the material is represented by σ_p_ − σ_exp_. Among these, DRX softening is the predominant type of softening observed in AZ91D magnesium alloys, and its extent can be quantified as a percentage:

(25) $$X_{drx}=\frac{\sigma_{p}-\sigma_{\exp}}{\sigma_{s}-\sigma_{ss}}$$

Jonas highlighted the determination of *σ*_s_ and *σ*_ss_ values from the *θ–σ* curve. Using 648 K/0.001s^−1^ as a reference, a tangent line is applied to the *θ–σ* curve with *σ*_c_ as the reference point. The intersection of this tangent line with θ = 0 yields the value of *σ*_s_, while the quadratic intersection of the *θ–σ* curve with θ = 0 provides the value of σ_ss_ (Figure 9b). Considering various *X_drx_* values and utilizing Equation (25), σ_exp_ can be determined, with corresponding strains obtained from Figure 2 where *ε* = *ε*_0.5_ at *X_drx_* = 50%. It is evident that the true stress–strain curve of the AZ91D magnesium alloy adheres to a distinct DRX pattern, with negligible influence. Combining with Equation (24), a linear fitting curve of ln[−ln(1 − *X_drx_*)] − ln[(*ε* − *ε*_c_)/*ε*_0.5_] is plotted in Figure 9c, illustrating an excellent linear fitting effect.

According to Equation (24), one can conclude that the *k_d_*-value and *β_d_*-value is 2.38672 and 1.88619, so one can obtain the DRX model:

(26) $$X_{drx}=1-\exp\left[-1.88619{\left(\frac{\varepsilon -0.66208\varepsilon_{p}}{\varepsilon_{0.5}}\right)}^{2.38672}\right]$$

According to the foregoing, one can obtain Equation (27):

(27) $$\ln\varepsilon_{0.5}=\ln a_{5}+m_{5}\ln\dot{\varepsilon }+\frac{Q_{drx}}{RT}$$

From Equation (27), ln*ε*_0.5_ is linearly related to $\ln\dot{\varepsilon }$ and 1000/*T*; one can obtain *m*_5_ = 0.09446, *Q*_5_/*R* = 4.19261 and ln*A*_5_ + *m*_5_ln$\dot{\varepsilon }$ = −7.61289, so *Q*_5_ = 34,857.35954 J/mol, and *A*_5_ = 4.94040 × 10^−4^. And one can obtain the *ε*_0.5_ for the AZ91D magnesium alloy:

(28) $$\varepsilon_{0.5}=4.94040\times 10^{-4}{\dot{\varepsilon }}^{0.09446}\exp\left(34857.35954/RT\right)$$

**Figure 10:**
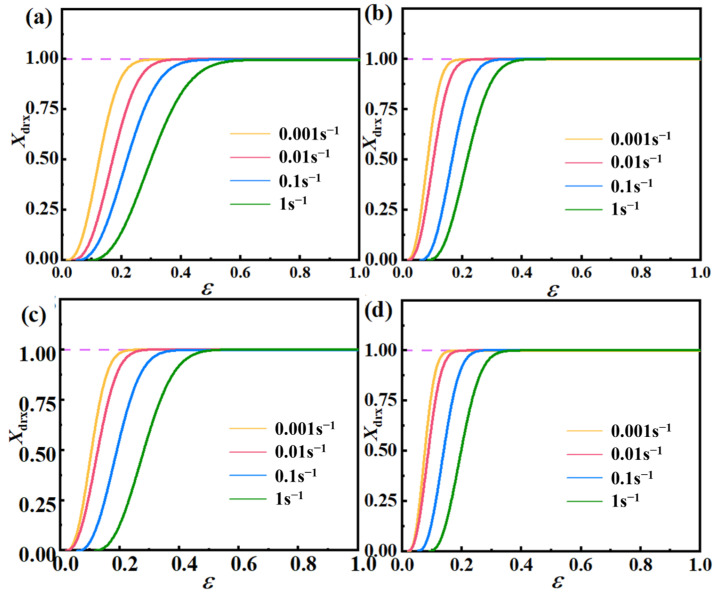
DRX volume fraction versus strain for different deformation temperature: (**a**) 648 K; (**b**) 673 K; (**c**) 698 K; (**d**) 723 K.

To explore the correlation between DRX volume fraction and strain, this study examined the relationship between *X_drx_* and *ε*, as defined in Equation (23). Figure 10 illustrates that the change in *X_drx_* can be categorized into three distinct stages as *ε* increases. At first, the rise in *X_drx_* is gradual, making it increasingly challenging to initiate dislocation slip, requiring higher external forces for DRX. Subsequently, *X_drx_* exhibits a rapid increase with rising *ε*, emphasizing the significant role of stress in driving DRX. Finally, the rate of *X_drx_* increase slows down, reaching its maximum value. Importantly, the stress needed to initiate DRX and reach this maximum value decreases with elevated temperature. This temperature-induced effect on additional dislocation generation plays a pivotal role in reducing the critical stress. Higher temperatures facilitate DRX occurrence in AZ91D magnesium alloy at lower critical values, making DRX more likely to happen.

According to Gourdet and Montheillet [38], the DRX grain size is mainly influenced by the original grain size, amount of deformation, strain rate, and alloy’s deformation activation energy. As a result, the DRX average grain size can be modeled:

(29) $$d_{drx}=a_{8}d_{0}^{h_{8}}\varepsilon^{n_{8}}{\dot{\varepsilon }}^{m_{8}}\exp\left(Q_{8}/RT\right)+c_{8}$$

where *d_drx_* is the average grain size, *d*_0_ is the original grain size, *Q*_8_ is the deformation activation energy, *a*_8_, *h*_8_, *n*_8_, *m*_8_, and *c*_8_ is the material correlation coefficient.

Since *d*_0_ is constant, *a*^8^d_0_*^h^*^8^*ε^n^*^8^ can be viewed as a constant *a*_8_, and taking logarithms of Equation (29) yields:

(30) $$\ln d_{drx}=\ln a_{8}+m_{8}\ln\dot{\varepsilon }+Q_{8}/RT$$

**Figure 11:**
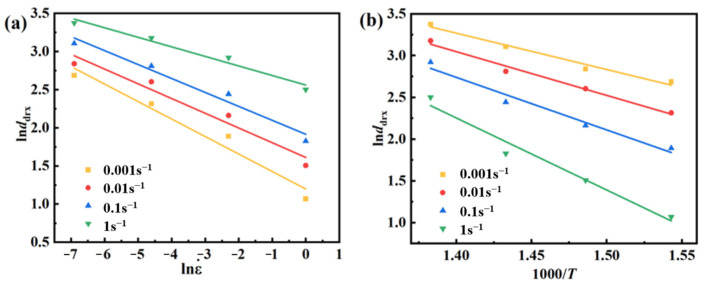
Linear fit between: (**a**) ln*d_drx_* − ln$\dot{\varepsilon }$ (**b**) ln*d_drx_* − 1000/*T*.

From Equation (30), one can see that ln*d_drx_* and ln$\dot{\varepsilon }$, ln*d_drx_* and 1000/*T* are linearly related. From the plots in Figure 11, one can find *m*_8_ = −0.18248, *Q*_8_ = −50,940.48 J/mol, and *a*_8_ = 713.21292.

Bringing the derived conclusion into Equation (29), one can derive the DRXed grain evolution model of AZ91D magnesium alloy as:

(31) $$d_{drx}=713.21292{\dot{\varepsilon }}^{-0.18248}\exp\left(-50940.48/RT\right)$$

### 3.4. Analysis of Microstructural Evolution

#### 3.4.1. Grain Size under Different Deformation Conditions

**Figure 12:**
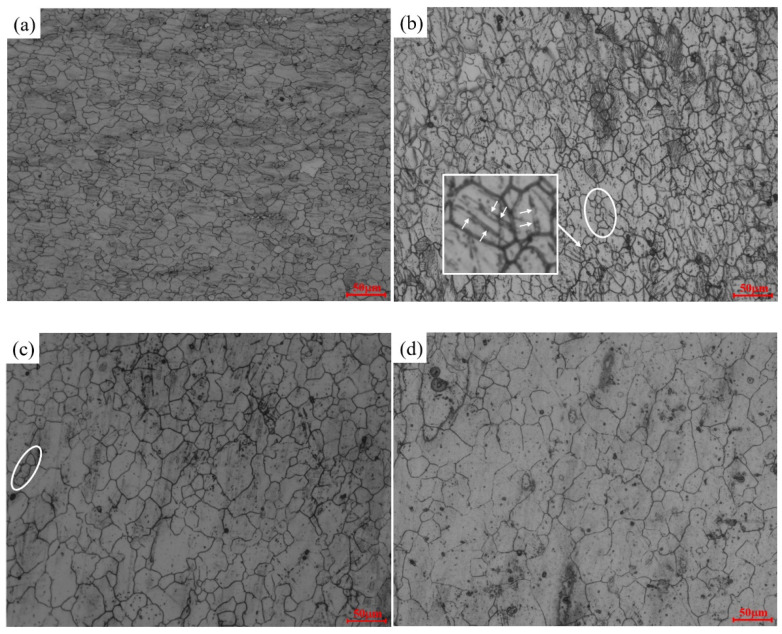
Microstructure of compressed alloy at a strain rate of 0.001 s^−1^: (**a**) 648 K; (**b**) 673 K; (**c**) 698 K; (**d**) 723 K.

Figure 12 depicts the microstructure of AZ91D magnesium alloy under a strain rate of 0.001 s^−1^. Notably, an increase in temperature corresponds to a rise in the average grain size. Focusing on the highlighted area within the figure, it becomes evident that the initiation of DRX in AZ91D magnesium alloy primarily takes place at the grain boundaries. The heightened deformation temperature serves as the driving force for DRX, subsequently triggering dislocation slip and twinning. Moreover, it facilitates nucleation and migration at the grain boundaries, collectively fostering the initiation and growth of DRX and leading to an enlargement of the average grain size. Additionally, In Figure 12b,c, the white circles indicate newly produced recrystallized grains, and the arrows in the square enlargement indicate the production of twins, which play a pivotal role in the deformation of magnesium alloy, aiding in coordinating the process when dislocations impose limitations.

**Figure 13:**
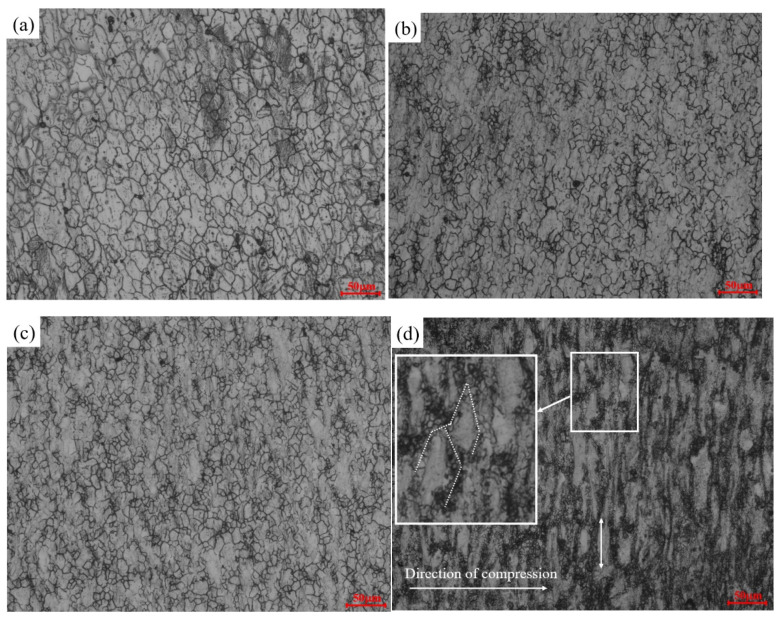
Microstructure of compressed alloy under the deformation temperature of 673 K: (**a**) 0.001 s^−1^; (**b**) 0.01 s^−1^; (**c**) 0.1 s^−1^; (**d**) 1 s^−1^.

Figure 13 illustrates the microstructure of AZ91D magnesium alloy under varying strain rates at 673 K. A clear trend emerges from the comparison of these figures: the average grain size diminishes with an increase in the strain rate. This outcome can be attributed to the heightened deformation rate, which reduces the available time for the deformation process. Consequently, DRX lacks sufficient time to exert its softening effect, and dislocation plugging at the grain boundaries is unable to facilitate adequate grain growth. As the strain rate escalates, the concentration of dislocation plugging stress intensifies, leading to elevated nucleation rates and an increased number of new nuclei. At excessively high strain rates, the distortion energy within the recrystallized grain rises. The dislocation density within the grain reaches the threshold for initiating a new round of recrystallization before the ongoing process completes, resulting in incomplete recrystallization, as evident in Figure 13d. The dashed line in the square localized magnification shows the new grains created around the original grains, and the double-arrowed straight line indicates that the grains are compressed in the sub-direction. In this incomplete recrystallization scenario, the original grains become flattened along the direction of hot compression, and numerous new nuclei form at the grain boundaries. Unfortunately, these new nuclei lack sufficient time to mature, as highlighted in the white box, illustrating a distinctive phenomenon arising from the constraints imposed by the rapid strain rate.

#### 3.4.2. Finite Element Response of a DRX Model with Experimental Verification

**Figure 14:**
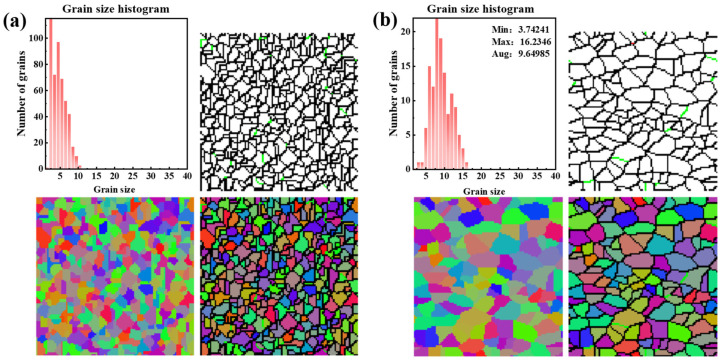
Microstructural chart of grain evolution at 0.1 s^−1^: (**a**) 648 K; (**b**) 673 K.

To validate the accuracy of the previously derived hot deformation constitutive equation and DRX model for AZ91D magnesium alloy, the acquired data were utilized in Deform-3D to analyze the evolution of grain size during the hot compression process. Figure 14a,b present simulated microstructure charts depicting grain evolution under 0.1 s^−1^ at 648 K and 673 K, respectively. It is crucial to note that this evolution reflects the general characteristics of the grains and does not signify changes in the original grains. The different colors are intended to distinguish the grains and do not represent grain orientation. A comparative analysis of these figures reveals that with an increase in the deformation temperature, the grain size also enlarges, in accordance with the fundamental law governing grain evolution. Moreover, their grain sizes are approximately 4.78 µm and 9.65 µm, which closely align with the grain sizes listed in Table 3 (5.62 µm and 8.69 µm). Table 3 presents the average grain size of the alloy as predicted by Deform-3D simulation (marked S) and as observed in the metallographic experiment (marked E). A comparison of these values indicates that the difference between the experimental and predicted results is minimal. This demonstrates that the DRX model employed in this study is highly accurate and capable of effectively predicting the DRX behavior.

**Figure 15:**
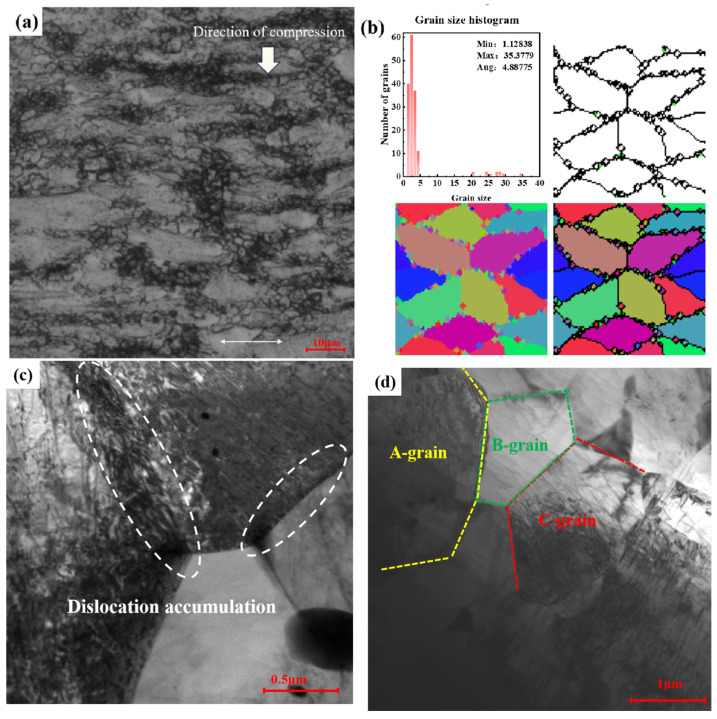
Microstructure of the alloy compressed at 673 K with 1 s^−1^: (**a**) actual metallograph; (**b**) simulated microstructure; (**c**) grain boundaries of recrystallized grains; (**d**) recrystallized grains.

Figure 15 displays the microstructure for the compressed alloy with 673 K/1 s^−1^. It is evident from this graph that the simulation results closely align with the actual observations, as shown in Figure 15a,b. Statistical analysis reveals that the grain size is approximately 4.89 µm, a value very close to the experimentally determined (4.51 µm) obtained from metallographic experiments. This close correspondence underscores the high accuracy of the model employed in this study. To explore the evolution process of recrystallized grains, TEM canonical analysis was performed on the grain boundaries of the alloy’s recrystallized grains. As illustrated in Figure 15c, throughout the deformation process, the alloy’s coarse grains initiate fracture owing to deformation. Localized regions of grain boundaries accumulate a significant number of dislocations, denoted by arrows, due to large strain energy. In this energetically unstable state, certain dislocations undergo rearrangement, forming new dislocation networks and walls proximal to grain boundaries through spontaneous interactions. Concurrently, some dislocation walls gradually transform into subgrain boundaries via migration and merging, giving rise to subgrains. Owing to the high energy and instability of grain boundaries in subgrains, as deformation progresses, there is a propensity for subgrains to merge and rotate, resulting in the formation of new undistorted grains, termed DRX grains. Figure 15d presents an enlarged microstructure image of the region captured under deformation conditions in Figure 15a. Notably, small grains proximate to the larger ones, as indicated, can be observed. In Figure 15d, marked as A-grain, are small DRXed grains forming within areas of pronounced aggregation. This observation suggests a correlation between the nucleation of DRXed grains and the continuous absorption of dislocations along these subgrain boundaries, categorizing the mechanism as CDRX. Correspondingly, in the figure, distinctly labeled as B-grain and C-grain, are typical DRXed grains that are entirely isolated and enclosed by subgrain boundaries, further substantiating that an in situ transformation from subgrains to DRXed grains can be achieved by the continuous increment of large-angle grain boundaries.

## 4. Conclusions

In this study, a comprehensive analysis of the flow stress characteristics and microstructure evolution of semi-solid forged AZ91D magnesium alloy is conducted through experimental and simulation technique. This approach serves to validate the model’s accuracy and visualize the changing parameters. The integrated system, encompassing physical experimentation, an ontological model, DRX behavior, and finite element simulation, underscores the significance of optimizing the forming process, controlling grain size, and enhancing overall performance. The key conclusions derived from the investigation are outlined as follows:

(1) The semi-solid forged AZ91D magnesium exhibits a DRX softening mechanism during high-temperature deformation. The rheological stress of the alloy decreases with increasing deformation temperature and decreasing strain rate. And activation energy is calculated to be 156.814 kJ/mol; the constitutive equation for the alloy is:

$$\sigma =\frac{1}{0.02857}\ln\left\{{\left(\frac{Z}{1.33208\times 10^{10}}\right)}^{\frac{1}{3.61637}}+{\left[{\left(\frac{Z}{1.33208\times 10^{10}}\right)}^{\frac{1}{3.61637}}+1\right]}^{\frac{1}{2}}\right\}$$

where:

$$Z=\dot{\varepsilon }\exp\left(156814/RT\right)$$

(2) When the semi-solid forged AZ91D alloy reaches the critical strain (ε_c_), dislocation accumulation reaches a critical value, leading to the occurrence of DRX. The critical strain model reflects the difficulty level of DRX. The DRX volume fraction indicates the progress of the alloy’s DRX process and determines the ultimate quality of the alloy’s mechanical properties. The grain size model reflects the growth trend of DRX grains. The DRX model of the semi-solid forged AZ91D magnesium alloy is calculated.

Critical strain model:

$$\varepsilon_{p}=0.004569{\dot{\varepsilon }}^{0.2662}\exp\left(20514.87814/RT\right)\;\sigma_{c}=0.92915\sigma_{p}\;\varepsilon_{c}=0.66208\varepsilon_{p}$$

Volume fraction model:

$$X_{drx}=1-\exp\left[-1.88619{\left(\frac{\varepsilon -0.66208\varepsilon_{p}}{\varepsilon_{0.5}}\right)}^{2.38672}\right]\;\varepsilon_{0.5}=4.94040\times 10^{-4}{\dot{\varepsilon }}^{0.09446}\exp\left(34857.35954/RT\right)$$

Grain size model:

$$d_{drx}=713.21292{\dot{\varepsilon }}^{-0.18248}\exp\left(-50940.48/RT\right)$$

(3) Under a constant strain rate of 0.001 s^−1^, elevated temperatures corresponded to larger average grain sizes. This effect was driven by increased deformation temperatures, creating favorable conditions for DRX. The higher temperature also activated intergranular dislocation slipping and twinning, promoting DRX nucleation and growth at grain boundaries, ultimately leading to larger average grain sizes. At a consistent temperature of 400 °C, an increase in strain rates resulted in a reduction of the average grain size. Higher deformation rates limited the available time for complete DRX softening, impeding grain growth at grain boundaries. However, higher strain rates induced stress concentration due to dislocation plugging, resulting in increased nucleation rates and the formation of more new nuclei. Extremely high strain rates caused original grains to flatten along the direction of hot compression, generating numerous new nuclei at grain boundaries, albeit with insufficient time for their growth.

(4) The average grain size, obtained through calculated equations, was integrated into Deform-3D to construct a material library for simulating the hot compression process. This simulation-based grain size was then compared with experimental results, thereby confirming the precision and accuracy of the calculated constitutive equations and DRX model for AZ91D magnesium alloy.

## Figures and Tables

**Table 1:** Main chemical composition of AZ91D magnesium alloy (mass fraction, %).

Al	Zn	Mn	Si	Cu	Fe	Ni	Mg
8.952	0.686	0.220	0.024	0.002	0.003	0.001	margin

**Table 2:** Physical parameters of AZ91D magnesium alloy in Deform-3D [12].

Parameters	Young’s Modulus	Poisson’s Ratio	Thermal Diffusion Coefficient	Radiation Intensity	Temperature Parameters
Value	50,500	0.34	2.65 × 10^−5^	0.7	250

**Table 3:** Recrystallized grain size under different deformation conditions, µm (S: Simulation, E: Experiment).

$\dot{\varepsilon }$	Temperature							
	648 K		673 K		698 K		723 K	
	S	E	S	E	S	E	S	E
0.001 s^−1^	13.24	14.67	16.88	17.12	20.51	22.33	27.45	29.21
0.01 s^−1^	8.85	9.11	12.49	13.51	14.21	16.62	22.51	23.97
0.1 s^−1^	4.78	5.62	9.65	8.69	10.88	11.51	18.66	18.54
1 s^−1^	3.56	2.91	4.88	4.51	6.33	6.21	13.89	12.18

## Data Availability

The original contributions presented in the study are included in the article, further inquiries can be directed to the corresponding author.

## References

[1] Nguyen T.L., Cheng T.C., Yang J.Y., Pan C.J., Lin T.H. (2022). A zinc-manganese composite phosphate conversion coating for corrosion protection of AZ91D alloy: Growth and characteristics. J. Mater. Res. Technol..

[2] Li M.X., Guo Q.W., Chen L.W., Li L.M., Hou H., Zhao Y.H. (2022). Microstructure and properties of graphene nanoplatelets reinforced AZ91D matrix composites prepared by electromagnetic stirring casting. J. Mater. Res. Technol..

[3] Tighiouaret S., Hanna A., Azzeddine H., Rabahi L., Dakhouche A., Brisset F., Helbert A.L., Baudin T., Bradai D. (2022). On the Evolution of microstructure, texture and corrosion behavior of a hot-rolled and annealed AZ31 alloy. Mater. Chem. Phys..

[4] Liang J.W., Lei Z.L., Chen Y.B., Wu S.B., Chen X., Jiang M., Cao S.Y. (2022). Formability, microstructure, and thermal crack characteristics of selective laser melting of ZK60 magnesium alloy. Mater. Sci. Eng. A.

[5] Yamagishi K., Ogawa Y., Ando D., Sutou Y. (2023). Adjustable room temperature deformation behavior of Mg-Sc alloy: From superelasticity to slip deformation via TRIP effect. J. Alloys Compd..

[6] Liu W., Wu B.Q., Liu H.R., Liu R.S., Mo Y.F., Tian Z.A., Hou Z.Y., Xi T.F., Wan Z.Y., Huang C.X. (2022). Simulation on microstructure evolution and mechanical properties of Mg-Y alloys: Effect of trace Y. Trans. Nonferr. Metal. Soc..

[7] Paramatmuni C., Bandi A., Kanjarla A.K. (2023). An experimental and crystal plasticity investigation of anisotropic compression behaviour of Mg-Sn-Ca alloy. J. Alloys Compd..

[8] Wang G.X., Mao P.L., Wang Z., Zhou L., Wang F., Liu Z. (2022). High strain rates deformation behavior of an as-extruded Mg-2.5Zn-4Y magnesium alloy containing LPSO phase at high temperatures. J. Mater. Res. Technol..

[9] Khan A.S., Baig M. (2011). Anisotropic responses, constitutive modeling and the effects of strain-rate and temperature on the formability of an aluminum alloy. Int. J. Plast..

[10] Zhao M.J., Huang L., Li C.M., Sun Y., Guo S.Q., Li J.J., Sun C.Y., Li P.C. (2023). Flow stress characteristics and constitutive modeling of typical ultrahigh-strength steel under high temperature and large strain. Steel Res. Int..

[11] Singh A.K., Narasimhan K. (2022). Determination and predication of formability on 22MnB5 steel under hot stamping conditions using Gleeble. Adv. Mater. Process. Technol..

[12] Sellars C.M., McTegart W.J. (1966). On the mechanism of hot deformation. Acta Metall..

[13] Duan W.X., Liu J.J., Liu L.L., Gong B., Li P., Liu B.S. (2019). Study on constitutive model and a model of continuous dynamic recrystallization softening behavior of AZ80A magnesium alloy. Mater. Res. Express.

[14] Luan J., Sun C.Y., Li X., Zhang Q. (2014). Constitutive model for AZ31 magnesium alloy based on isothermal compression test. Mater. Sci. Technol..

[15] Chen B., Zhou W.M., Li X.L., Lu C. (2013). Optimization of hot extrusion process parameters of Mg97Y2Zn1 alloy based on the processing maps. J. Mater. Eng. Perform..

[16] Galiyev A.M., Kaibyshev R.O., Gottstein G. (2002). Grain refinement of ZK60 magnesium alloy during low temperature deformation. Acta Mater..

[17] Tan J.C., Tan M.J. (2003). Dynamic continuous recrystallization characteristics in two stage deformation of Mg-3Al-1Zn alloy sheet. Mater. Sci. Eng. A.

[18] Mirzadeh H. (2023). Grain refinement of magnesium alloys by dynamic recrystallization (DRX): A review. J. Mater. Res. Technol..

[19] Barrett C.D., Imandoust A., Oppedal A.L., Inal K. (2017). Effect of grain boundaries on texture formation during dynamic recrystallization of magnesium alloys. Acta Mater..

[20] Nakata T., Xu C., Geng L., Kamado S. (2022). Twinning-mediated texture weakening in a basal-textured Mg-6Al-1Zn (mass%) alloy sheet by a novel cold-sample rolling method. J. Alloys Compd..

[21] Tang T., Shao Y.C., Li D.Y., Peng L.M., Peng Y.H., Zhang S.R., Wu P.D. (2018). Polycrystal plasticity simulation of extrusion of a magnesium alloy round bar: Effect of strain path non-uniformity. J. Alloys Compd..

[22] Kasaeian-Naeini M., Sedighi M., Hashemi R. (2022). Severe plastic deformation (SPD) of biodegradable magnesium alloys and composites: A review of developments and prospects. J. Magnes. Alloy..

[23] Jiang M.G., Xu C., Nakata T., Yan H., Chen R.S., Kamado S. (2017). Enhancing strength and ductility of Mg-Zn-Gd alloy via slow-speed extrusion combined with pre-forging. J. Alloys Compd..

[24] Jia W.T., Le Q.Z., Tang Y., Ding Y.P., Ning F.K., Cui J.Z. (2018). Role of pre-vertical compression in deformation behavior of Mg alloy AZ31B during super-high reduction hot rolling process. J. Mater. Sci. Technol..

[25] Mroczka K., Dymek S., Węglowska A., Hamilton C., Kopyściański M., Pietras A., Kurtyka P. (2023). Comprehensive research of FSW joints of AZ91D magnesium alloy. Materials.

[26] Xu S.W., Zhu C.C., Kamado S., Oh-ishi K., Qin Y. (2022). Dynamic recrystallization behavior of as-cast AZ91 magnesium alloy during hot compressive. J. Mater. Res. Technol..

[27] Luo A., Pekguleryuz M.O. (1994). Cast magnesium alloys for elevated temperature applications. J. Mater Sci..

[28] Long J.C., Xia Q.X., Xiao G.F., Qin Y., Yuan S. (2021). Flow characterization of magnesium alloy ZK61 during hot deformation with improved constitutive equations and using activation energy maps. Int. J. Mech. Sci..

[29] McQueen H.J., Ryan N.D. (2002). Constitutive analysis in hot working. Mater. Sci. Eng. A.

[30] Li Y., Hou P.J., Wu Z.G., Ren Y., Choo H. (2021). Dynamic recrystallization of a wrought magnesium alloy: Grain size and texture maps and their application for mechanical behavior predictions. Mater. Charact..

[31] Yin L., Wu Y.X. (2022). Comparison of constitutive models and microstructure evolution of GW103K magnesium alloy during hot deformation. Materials.

[32] Guo W.M., Li N., Zhou J.X., Liu L., Tian L.N., Chen L.Z., Zaïri F., Ding N. (2021). Flow curve and microstructure analysis of a ZK60 magnesium alloy during hot compression tests. Metallogr. Microstruct..

[33] Sellars C.M., Tegart W.J. (1966). The relationship between the resistance and the structural deformation in the hot, memories scientific review. Acta Metall. Sin..

[34] Ullmann M., Kittner K., Prahl U. (2021). Hot deformation and dynamic recrystallisation behaviour of twin-roll cast Mg-6.8Y-2.5Zn-0.4Zr magnesium alloy. Materials.

[35] Najafizadeh A., Jonas J.J., Stewart G.R., Poliak E.I. (2006). The strain dependence of postdynamic recrystallization in 304 H stainless steel. Metall. Mater. Trans. A.

[36] Sun Y.H., Wang R.C., Ren J., Feng Y. (2019). Hot deformation behavior of Mg-8Li-3Al-2Zn-0.2Zr alloy based on constitutive analysis, DRX kinetics, and processing map. Mech. Mater..

[37] Wu H., Xu W.C., Wang S.B., Yang Z.Z., Chen Y., Teng B.G., Shan D.B., Guo B. (2020). A cellular automaton coupled FEA model for hot deformation behavior of AZ61 magnesium alloys. J. Alloys Compd..

[38] Gouedet S., Montheillet F. (2003). A model of continuous dynamic recrystallization. Acta Mater..

